# Tracking quality of life-related outcomes in the U.S. population with monthly PROMIS computerized adaptive testing

**DOI:** 10.1007/s11136-026-04279-9

**Published:** 2026-06-06

**Authors:** D. U. Junghaenel, S. Schneider, A. A. Stone, B. Orriens, T. Gutsche, J. Darling, F. Perez-Arce, O. Hayden, A. Kapteyn

**Affiliations:** 1 Center for Economic & Social Research, University of Southern California, Los Angeles, CA USA; 2 Department of Psychology, University of Southern California, Los Angeles, CA USA

**Keywords:** Patient-reported outcomes, Population health, Intensive monitoring, Understanding America Study

## Abstract

**Purpose:**

This study evaluated the feasibility, reliability, and sensitivity of repeatedly administering brief PROMIS® computerized adaptive tests (CATs) monthly in a large, nationally representative internet panel.

**Methods:**

PROMIS CATs for anger, meaning in life, and positive affect were administered monthly for 13 consecutive months to the same 12,231 U.S. adults in the Understanding America Study (UAS). Each CAT was limited to three items per domain to minimize respondent burden. We assessed measurement precision, rank-order stability, and predictors of overall levels and month-to-month variability in each PROMIS measure. Time-series analyses also tested whether PROMIS scores were sensitive to acute health events reported each month.

**Results:**

Three-item CATs achieved strong measurement precision (reliability ≥ 0.80) across wide score ranges and showed moderate rank-order stability (ICCs = 0.67–0.79). In between-subjects analyses, a greater number of chronic conditions was associated with both poorer average PROMIS scores and higher month-to-month variability. In within-subjects analyses, PROMIS scores were responsive to acute health events: anger and positive affect showed significant same-month changes in response to most health events, while meaning in life was less reactive. Event-related changes typically dissipated within 1–2 months.

**Conclusions:**

Brief PROMIS CATs can be administered monthly while maintaining psychometric rigor. This high-frequency approach enables the assessment of both average levels and temporal dynamics of health, revealing new insights into the effects of chronic disease and acute health events. These findings support the use of PROMIS measures in longitudinal population health research.

**Supplementary Information:**

The online version contains supplementary material available at 10.1007/s11136-026-04279-9.

## Introduction

The Patient-Reported Outcomes Measurement Information System (PROMIS®) is a set of rigorously developed instruments to assess key domains of health-related quality of life (QoL) [1, 2]. Developed through a National Institutes of Health (NIH) initiative, PROMIS measures are grounded in item response theory (IRT) and calibrated to a common metric, facilitating comparability across populations and studies. One of the central innovations of PROMIS is its use of computerized adaptive testing (CAT), which dynamically selects the most informative items based on each respondent’s prior answers. This adaptive approach substantially reduces respondent burden while preserving high precision across the continuum of each construct [3, 4]. PROMIS CATs are now widely used across clinical and research settings, including monitoring of patient-reported outcomes in oncology, rehabilitation, primary care, and mental health [5–8]. Their increasing adoption in health systems and large cohort studies reflects their flexibility, psychometric rigor, and responsiveness to a wide range of health and life circumstances.

Although PROMIS was originally developed to assess a wide range of health domains, many of its measures focus on aspects of emotional and psychosocial functioning that are relevant to both clinical and general population research [6, 8]. The present study focuses on three such domains—anger, positive affect, and meaning in life—which reflect core dimensions of well-being. These outcomes are particularly relevant for large-scale monitoring, as they are influenced by both chronic health conditions and acute life events. Although these domains differ in expected time-scale of change—with anger and positive affect typically more reactive and meaning in life more stable—they each offer distinct insight into psychological functioning and health-related adaptation.

### High frequency monitoring with PROMIS measures in the general population

To date, most research using PROMIS has relied on cross-sectional assessments or infrequent longitudinal designs. While such designs are appropriate for many evaluative purposes, they do not fully capture the dynamic nature of psychological well-being as people move through life’s events and transitions. High-frequency assessment—such as monthly measurement over extended periods—offers several important advantages for population-based research [2, 9, 10]. First, it enables ongoing public health monitoring by tracking outcomes at the population level in near-real time. Just as economic indicators are monitored continuously to inform policy, regular PROMIS assessments could help identify emerging trends and disparities in psychosocial functioning and well-being. Second, high-frequency data allow researchers to examine not only average levels of psychological well-being, but also *how much people’s experiences fluctuate over time*. A substantial body of research spanning psychology, psychiatry, and aging has shown that individuals differ in their degree of intraindividual variability, and that this variability itself can be an important health marker. For example, greater variability in emotions, mood, and well-being have been linked to psychological distress and greater health problems [11, 12]. Finally, monthly assessment enhances sensitivity to systematic short-term changes in psychological well-being following health-related events or life disruptions, such as illness, injury, or diagnosis of a disease. This supports a more nuanced understanding of how individuals recover or adapt to change, an area of increasing relevance for both clinical and population health research.

Against this backdrop, internet-based panel studies offer an important platform for large-scale, high-frequency population monitoring. Unlike traditional panel studies conducted via in-person interviews or telephone, online panels provide greater flexibility in administration, reduced logistical and administrative burden, and the opportunity to implement computerized adaptive testing (CAT) in a standardized and efficient manner.

### The present study

This study aims to evaluate the reliability, validity, and sensitivity of these monthly PROMIS CAT assessments in a general population context using data from the Understanding America Study (UAS), a nationally representative internet-based panel of the general U.S. adult population.

First, we address concerns about respondent burden in high-frequency data collection. Although PROMIS CATs are known for their efficiency and precision, standard recommendations typically call for four to seven items per administration [2]. To minimize respondent burden, we aimed to evaluate whether very brief monthly PROMIS CATs (limited to three items per domain), would still yield acceptable levels of measurement precision for population-based research. In addition to measurement precision, monthly assessments also allow us to evaluate whether the measures preserve individuals’ relative standing over time—a psychometric form of stability known as *rank-order stability*. This provides a longitudinal index of score reliability, not an expectation that individuals themselves remain stable across months.

Second, we examine between-person differences in average levels and in month-to-month variability in each PROMIS outcome. Average levels and the amount of variability represent distinct aspects of psychological functioning. We hypothesized that respondents with more chronic health conditions reported prior to the monthly assessments would report lower average levels of positive affect and meaning in life, and higher average levels of anger. These expectations are grounded in existing evidence linking multimorbidity to poorer QOL as shown in prior work on PROMIS [6, 13]. Moreover, individuals with worse health may face more frequent or unpredictable stressors, which could contribute to greater intraindividual variability in emotional states across time [12, 14]. Thus, we also expected that individuals with more chronic conditions would show stronger month-to-month fluctuations in psychological well-being.

Third, we assess the sensitivity of PROMIS CAT scores to acute health events. Prior research shows that adverse health events—including illness, injury, and new diagnoses—can influence emotional well-being [15]. Accordingly, we expected the three PROMIS domains to show within-person changes following health disruptions. Demonstrating such responsiveness provides evidence of sensitivity to change and clarifies the time-scale on which different domains of psychological well-being react to health-related stressors.

## Methods

### Study design and participants

Data for the current study were collected in the UAS, a nationally representative internet panel administered by the University of Southern California [16, 17]. Unlike opt-in panels, which rely on self-selected respondents, the UAS recruits members using address-based sampling to ensure probability-based representation. This approach uses a sampling frame derived from the U.S. Postal Service list of residential addresses, helping to mitigate common biases associated with nonprobability samples [18, 19]. To ensure equitable participation, individuals without home internet are provided with a tablet and broadband connection—a key consideration given the lower rates of internet access among older adults and individuals with less formal education [20]. Panel members complete online surveys approximately once or twice per month on a wide range of topics. The respondents provided electronic informed consent for participation. The study was approved by the Biomedical Research Alliance of New York Institutional Review Board (BRANY IRB: 22-0301044).

Beginning in October 2023, all active members of the UAS panel were invited to complete brief monthly surveys, administered at the start of each month. These surveys included questions about life events experienced during the prior month. PROMIS measures of anger, meaning in life, and positive affect were introduced in March 2024, and the present analyses include data from the 13 monthly surveys conducted between March 2024 and March 2025. All measures were repeatedly administered to the same panel members. We excluded respondents who completed only one wave (*n* = 651; 5.05%) to ensure a sufficient basis for modeling within-person change over time.

### Measures

PROMIS has developed calibrated item banks to assess key domains of psychosocial functioning, including anger (22 items) [21], meaning in life (37 items) [22], and positive affect (34 items) [23]. Items are calibrated using a T-score metric, anchored to a mean of 50 and standard deviation of 10 in the U.S. general population [2]. PROMIS CATs were administered using the R mirtCAT package [24] via the NubiS platform, an online survey environment developed for data collection in the UAS. The CAT implementation followed standard PROMIS procedures: provisional theta estimates were initialized at 0, scoring used an EAP estimator, and items were selected iteratively using the Maximum Posterior Weighted Information (MPWI) criterion. The CAT was programmed to administer exactly three items, as prior work shows that most precision gains occur in the first few items and returns diminish rapidly thereafter [2].

Chronic health conditions are routinely reported by all UAS respondents approximately every two years as part of a survey module using questions developed in the Health and Retirement Study (HRS). Respondents were asked “has a doctor ever told you that you have …?” Conditions were diabetes or high blood sugar, cancer or malignant tumor, lung disease such as chronic bronchitis or emphysema, heart disease (heart attack, coronary heart disease, angina, congestive heart failure), a stroke, and arthritis or rheumatism. For each respondent, we used the information from the most recent report prior to the monthly surveys and counted the number of health conditions. We then grouped respondents into those who reported zero chronic conditions, one condition, two conditions, three conditions, or more than three of the 6 conditions, following prior PROMIS validation research [6].

Questions about 19 acute health events occurring over the past month were asked at each monthly survey. These included accident or injury, assault, influenza, pneumonia, COVID-19, shingles, arthritis-related surgery or joint replacement, and new disease diagnoses: cancer, diabetes, heart-related conditions (e.g., heart attack), kidney disease, chronic lung disease (e.g., bronchitis or emphysema), arthritis or rheumatism, osteoporosis, dementia or serious memory impairment, high blood pressure or hypertension, emotional or psychiatric conditions, sleep disorder, and another illness not listed above.

### Analyses

A first analysis step examined the measurement precision of the three-item PROMIS computerized adaptive tests (CATs) administered for each health domain. IRT-based measures estimate scores with precision that varies across the severity continuum of the underlying construct, as captured by the test information function [1, 25]. The standard error of measurement (SE) for T-scores can be translated into reliability using the general IRT formulation: reliability = 1 – (SE²/Var(ϴ)), where Var(ϴ) denotes the variance of the latent trait. Because PROMIS T-scores are scaled to have a fixed SD of 10 in the reference population, this simplifies to reliability = 1 – (SE²/100). For population-level research, reliability coefficients exceeding 0.70 are generally considered acceptable, with 0.80 or higher preferred for distinguishing between individuals or tracking group-level trends over time.

Before evaluating predictors of within-person variability or responses to acute health events, we first characterized the longitudinal structure of each PROMIS domain. This descriptive step assesses how much of the total variability in monthly scores reflects stable between-person differences versus month-to-month fluctuations within persons. This approach does not assume that individuals are unchanging over time; rather, it quantifies the proportion of variance attributable to each level. A high proportion of between-person variance indicates greater rank-order stability, that is, individuals tend to maintain their relative standing compared to others, even as meaningful within-person changes may still occur.

To estimate these components, we fit unconditional multilevel models with monthly assessments nested within individuals. Rank-order stability was quantified using intraclass correlation coefficients (ICCs), calculated as ICC = between-person variance/(between-person variance + pooled within-person variance) [26]. Higher ICCs reflect greater persistence of individual differences over time, whereas lower ICCs reflect greater relative within-person fluctuation. In supplementary analyses, we also estimated ICCs separately for participants with and without chronic conditions by fitting the same unconditional random-intercept model within each subgroup.

Next, we examined whether respondents with more chronic health conditions (assessed with the HRS health questions) reported lower average levels and greater month-to-month variability in PROMIS scores. We used a multilevel location-scale model in which monthly PROMIS scores were nested within individuals. At Level 1, repeated PROMIS scores were modeled with a person-specific intercept (reflecting each individual’s average score) and residual variance (reflecting within-person variability), a first-order residual autoregressive term accounted for temporal dependencies. Unlike traditional multilevel models, which assume a single residual variance for all individuals, the location-scale model estimates both the mean levels (“location”) and the within-person residual variance (“scale”) as random effects. The scale component models individual-specific residual variances on the log scale, allowing each person to have a distinct degree of month-to-month variability [27, 28]. For descriptive purposes, we transformed the estimated log variances into within-person standard deviations (iSDs). At Level 2, both the location and scale components were regressed on respondents’ number of chronic health conditions (entered as a categorical predictor), controlling for age, gender, race, and education to account for known demographic health correlates. The coefficient predicting the location component indicates whether individuals with more chronic conditions report lower average PROMIS scores. The coefficient predicting the scale component indicates whether chronic conditions are associated with increased month-to-month variability in PROMIS scores.

Finally, we used multilevel time series models [28] to examine the within-person effects of acute health events on monthly PROMIS scores. The data form a monthly time series nested within individuals, and each acute health event was coded as a time-varying binary indicator (1 = event occurred during this month; 0 = did not occur during this month). These indicators were person-mean centered to isolate month-to-month deviations in health status from individuals’ overall likelihood of experiencing events, following best-practice recommendations for modeling within-person effects [29]. The centered event indicators were entered as Level-1 predictors. We tested both contemporaneous (“same-month” denoting a time lag of less than a month between the acute health event and the PROMIS assessment) and lagged effects (1- to 4-month lags). A first-order autoregressive term (AR [1]) accounted for temporal dependencies. Individuals who never experienced a given event naturally contributed no within-person variation for that predictor but remained in the model and contributed information to intercepts, autoregressive parameters, and covariates. Primary analyses examined the impact of any of 19 assessed health events, with supplemental analyses modeling each event type separately.

The analyses were conducted using a Bayesian estimator with default priors in M*plus* version 8.11 [30], interfaced through the R package *MplusAutomation* [31]. This approach incorporates all available data under the assumption of missing at random (MAR).

## Results

Of 16,129 eligible UAS panel members who were invited, 12,231 (75.8%) completed at least two monthly assessments and were analyzed (see Table 1). Compared to nonrespondents, respondents were significantly older and more likely to identify as non-Hispanic White, but showed similar distributions in gender, education, and income (Supplementary Table S1). On average, participants completed 10.38 of the 13 repeated monthly assessments (*SD* = 3.50; median = 12), yielding a total of 127,643 observations. The analyzed sample had a mean age of 50.94 years (*SD* = 16.31), with 61.2% identifying as female and 59.7% as non-Hispanic white. Most had some college education or higher (79.3%) and one-third reported annual household incomes of less than $50,000 (37.80%). Just over half of the sample (56.2%) reported no chronic conditions, while 25.5% reported one, and 11.7% reported two. Smaller proportions reported three (4.7%) or more than 3 (2.1%). The most common conditions included arthritis (27.2%), diabetes (18.4%), and heart disease (10.4%), see Table 1.

**Table 1:** Sample characteristics (*N* = 12,231)

	*n* (%) or Mean (SD)
*Age*	
Mean (*SD*) years	50.94 (*SD* = 16.31)
18–34 years	2287 (18.74%)
35–49 years	3579 (29.32%)
50–64 years	3389 (27.76%)
65 + years	2952 (24.18%)
Female	7482 (61.19%)
*Race/Ethnicity*	
Non-Hispanic White	7296 (59.72%)
Hispanic	1933 (15.82%)
Other or mixed	2989 (24.46%)
*Education*	
Mean (*SD*) years	14.73 (*SD* = 2.61)
High school or less	2536 (20.74%)
Some college	4107 (33.58%)
Bachelor’s degree or higher	5586 (45.68%)
*Annual Household Income*	
Less than $50,000	4613 (37.80%)
$50,000-$99,999	3609 (29.57%)
$100,000 or more	3981 (32.62%)
*Self-reported chronic conditions*	
Diabetes	2256 (18.44%)
Cancer	1064 (8.70%)
Lung disease	634 (5.18%)
Heart disease	1269 (10.38%)
Stroke	299 (2.44%)
Arthritis	3322 (27.16%)

Some frequencies sum to less than the total sample size due to missing values

### Measurement precision and rank-order stability

**Fig. 1:**
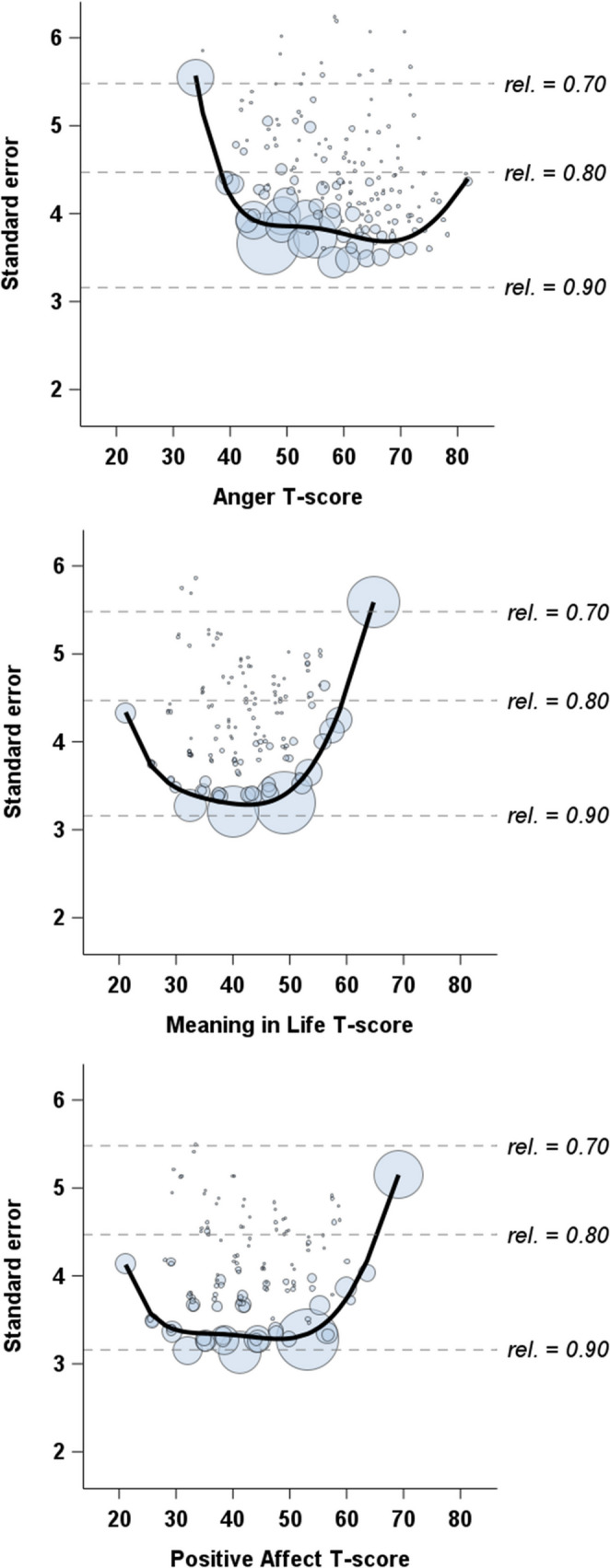
Estimated standard errors for monthly PROMIS T-scores of anger, meaning in life, and positive affect, based on three items administered via computerized adaptive testing. The size of the circles represents the relative frequency of scores. Solid lines represent fifth-order polynomial fits

Figure 1 displays the estimated standard errors for monthly T-scores in each domain, each based on three-item CATs. Reliabilities exceeded a 0.80 threshold across wide portions of the score continuum: from 40 to 80 for anger (covering 91% of all monthly T-scores), 20 to 60 for meaning in life (83% of scores), and 20 to 65 for positive affect (86% of scores). The average estimated reliabilities were 0.84 (median = 0.85, mode = 0.87) for anger, 0.85 (median = 0.88, mode = 0.89) for meaning in life, and 0.86 (median = 0.89, mode = 0.89) for positive affect. For descriptive context, we compared each 3-item CAT with the shortest available PROMIS fixed short form for its domain. Across domains, the CATs recovered a large proportion of the information contained in substantially longer fixed forms (see Supplementary Table S2). For each domain, we further summarized the frequency with which each item was administered and the mean theta level at which it was selected. As expected for adaptive testing, lower-scoring participants tended to receive easier items, whereas higher-scoring participants tended to receive more difficult items (Supplementary Table S3).

ICCs across monthly assessments were 0.67 for anger, 0.79 for meaning in life, and 0.70 for positive affect, suggesting moderate rank-order stability, with the highest stability observed for meaning in life. Approximately 33%, 21%, and 30% of the variance, respectively, reflected within-person fluctuations over time. When estimated separately for participants with and without chronic conditions, ICCs were similar in magnitude, indicating that both groups exhibited nontrivial month-to-month fluctuations in PROMIS levels (Supplementary Table S4). These values indicate both stable individual differences and meaningful month-to-month changes in PROMIS levels.

**Fig. 2:**
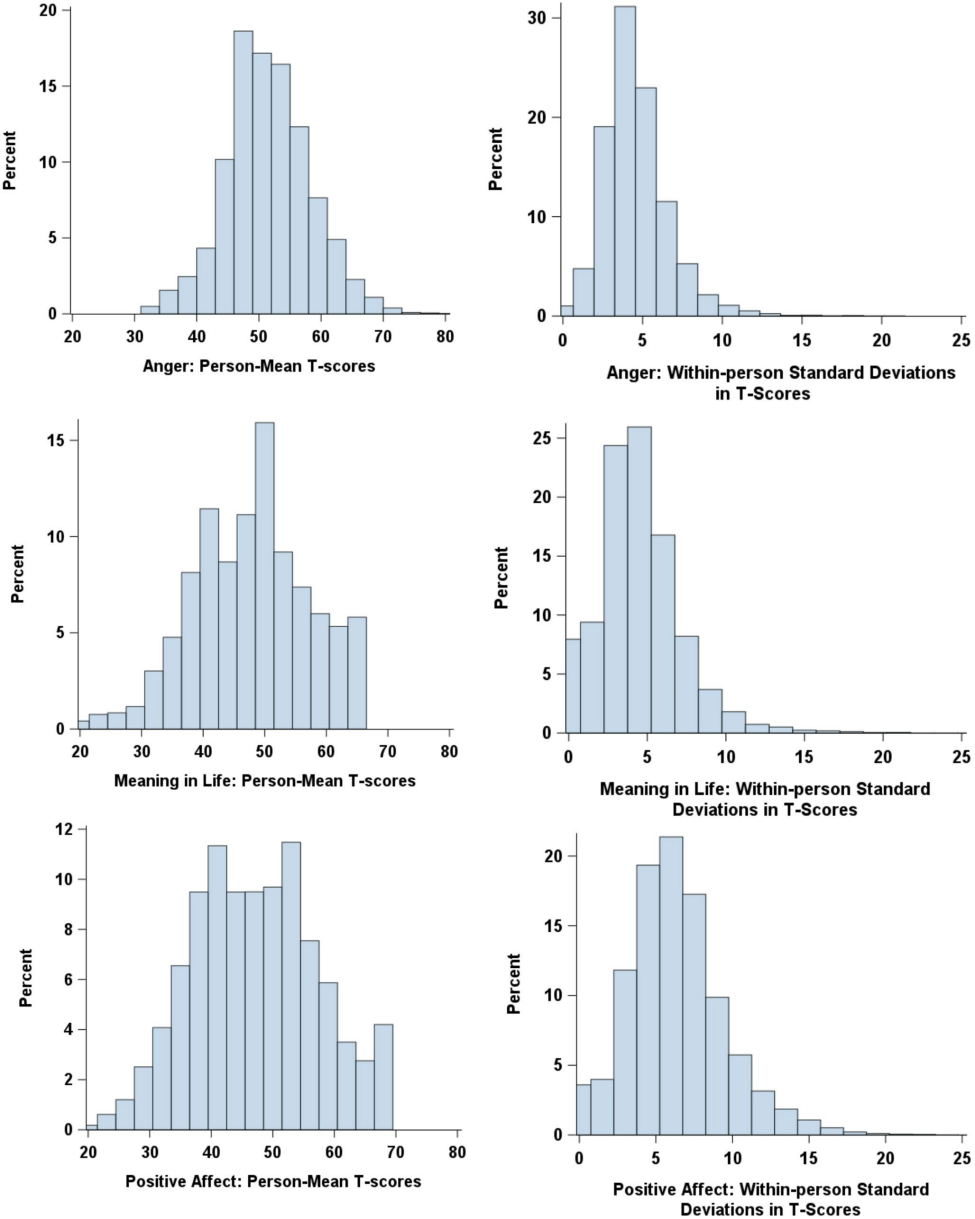
Histograms of respondents’ average levels and within-person variability in PROMIS scores. Values were derived from location-scale multilevel models. To aid interpretability, within-person variability is expressed as individual standard deviations, calculated by exponentiating and square-rooting the person-specific log variances

Figure 2 shows the distributions of respondents’ average levels and within-person variability in PROMIS scores, as estimated from multilevel location-scale models. For anger, the median intraindividual variability in iSD units was 4.49 T-score points, with the 10th and 90th percentiles at 2.39 and 7.08. For meaning in life, the median was 4.20, with a spread from 1.29 to 7.61. Positive affect showed the greatest variability, with a median iSD of 6.02 and a 10th–90th percentile range from 2.70 to 10.33. These results indicate that individuals differ not only in their average QoL levels, but also in how much their PROMIS scores fluctuate from month to month.

### Predictors of average levels and intraindividual variability in PROMIS scores

Regression results from the location-scale models predicting average levels and within-person variability in PROMIS scores are presented in Table 2. Older age was associated with significantly lower levels of anger, more meaning in life, more positive affect, and with significantly less month-to-month variability across all three PROMIS domains. Women reported less anger and more meaning in life, but showed greater within-person variability across all domains. White respondents reported less favorable average scores (more anger, less meaning, less positive affect) whereas higher education was associated with more favorable scores and lower variability in all domains.

**Table 2:** Multilevel regressions (95% credible intervals) predicting average levels and within-person variability in monthly PROMIS scores

Between-person predictors	Anger	Meaning in life	Positive affect
*Average PROMIS level*			
Intercept (no chronic condition)	50.68 (50.49, 50.84)	48.81 (48.56, 49.07)	48.65 (48.38, 48.93)
Age (years)	− 0.172 (− 0.180, − 0.163)	0.087 (0.074, 0.099)	0.091 (0.078, 0.104)
Female	− 0.979 (− 1.243, − 0.754)	0.904 (0.552,1.261,)	0.407 (− 0.003, 0.800)^a^
White race	0.852 (0.558, 1.140)	− 2.207 (− 2.593, − 1.787)	− 1.078 (− 1.507, − 0.611)
Education (years)	− 0.182 (− 0.229, − 0.141)	0.169 (0.104, 0.239)	0.274 (0.195, 0.351)
1 chronic condition	1.415 (1.117, 1.718)	− 1.555 (− 1.996, − 1.140)	− 2.475 (− 1.994, − 2.978)
2 chronic conditions	2.164 (1.744, 2.587)	− 2.861 (− 3.489, − 2.247)	− 4.014 (− 4.658, − 3.364)
3 chronic conditions	3.037 (2.431, 3.651)	− 4.158 (− 5.011, − 3.337)	− 5.740 (− 6.641, − 4.849)
>3 chronic conditions	3.823 (2.978, 4.774)	− 4.217 (− 5.515, − 2.953)	− 6.615 (− 7.938, − 5.130)
*Within-person variability*			
Intercept (no chronic condition)	2.753 (2.713, 2.786)	2.171 (2.104, 2.235)	3.189 (3.134, 3.242)
Age (years)	− 0.017 (− 0.018, − 0.015)	− 0.015 (− 0.018, − 0.012)	− 0.011 (− 0.014, − 0.008)
Female	0.119 (0.074, 0.162)	0.156 (0.062, 0.252)	0.288 (0.216, 0.367)
White	− 0.311 (− 0.361, − 0.253)	− 0.245 (− 0.347, − 0.137)	− 0.033 (− 0.118, 0.050)^a^
Education (years)	− 0.038 (− 0.048, − 0.029)	− 0.107 (− 0.125, − 0.091)	− 0.016 (− 0.029, − 0.002)^b^
1 chronic condition	0.140 (0.079, 0.204)	0.197 (0.085, 0.305)	0.140 (0.048, 0.234)
2 chronic conditions	0.196 (0.115, 0.272)	0.319 (0.174, 0.472)	0.197 (0.085, 0.326)
3 chronic conditions	0.252 (0.139, 0.362)	0.404 (0.190, 0.634)	− 0.089 (− 0.265, 0.094)^a^
>3 chronic conditions	0.400 (0.228, 0.578)	0.683 (0.348, 1.012)	0.410 (0.130, 0.668)

All regression coefficients are significant at *p* < 0.001 except ^a^*p* ≥ 0.05, ^b^*p* < 0.05, ^c^*p* < 0.01

Compared to respondents without chronic medical conditions, individuals with one or more conditions reported significantly higher anger, lower meaning in life, and less positive affect, controlling for demographic characteristics. For example, those with more than three conditions had anger scores nearly 4 T-score points higher, meaning in life scores about 4 points lower, and positive affect scores nearly 7 points lower than those without conditions. Respondents with chronic conditions also showed significantly greater month-to-month variability in each PROMIS domain. These associations generally followed a monotonic pattern, with each additional condition linked to incremental changes in both average levels and variability—except for positive affect variability, which did not increase for respondents reporting exactly three conditions. For example, respondents with more than three chronic conditions showed a 49% increase in within-person variance in anger scores relative to those without any chronic conditions (from 15.7 to 23.4 T-score squared units), corresponding to a 22% increase in iSDs (from 3.96 to 4.84). Meaning in life variability increased even more sharply—by 98% in within-person variance (from 8.8 to 17.4) and 41% in iSDs (from 2.96 to 4.18). For positive affect, within-person variance increased by 50% (from 10.2 to 15.3), reflecting a 22% increase in iSDs (from 3.20 to 3.91). These findings suggest that individuals with chronic health conditions not only report worse PROMIS scores on average, but also experience greater fluctuations in PROMIS scores over time.

We observed similar, though generally smaller, associations when examining specific health conditions individually (see Supplementary Table S5), suggesting that cumulative disease burden may better capture the processes driving the levels and fluctuations in psychological well-being.

### Responsiveness to acute health events

Table 3 summarizes results from multilevel time series models testing the within-person effects of acute health events on monthly PROMIS scores, adjusting for first-order autoregressive effects to account for carryover from prior months. The autoregressive parameters (AR [1]) ranged from 0.18 to 0.19 across outcomes, indicating modest temporal dependence in PROMIS scores. Health events were associated with significant shifts in PROMIS scores observed for the same month as the events: anger increased (b = 0.78, 95% CI [0.64, 0.91]), while meaning in life (b = − 0.31, 95% CI [–0.46, − 0.19]) and positive affect (b = − 1.70, 95% CI [–1.90, − 1.52]) decreased. One-month lagged effects were smaller and only significant for positive affect (b = − 0.37, 95% CI [–0.58, − 0.13]), with no consistent effects at longer lags. As a robustness check, we repeated the time-series analyses restricting the sample to individuals who experienced at least one acute health event. Effect sizes, autoregressive parameters, and residual variance estimates were substantively similar to those obtained in the full sample, indicating that the pooled results were not driven by heterogeneity between event and non-event participants (see Supplementary Table S6).

**Table 3:** Within-person effects (95% credible intervals) of health events on monthly PROMIS scores

Within-person predictors	Anger	Meaning in life	Positive affect
Autocorrelation AR(1)	0.183 (0.176, 0.190)***	0.194 (0.187, 0.201)***	0.180 (0.173, 0.187)***
Health event (same month)	0.781 (0.635, 0.910)***	− 0.313 (− 0.458, − 0.186)***	− 1.702 (− 1.898, − 1.523)***
Health event (1-month lag)	0.132 (− 0.031, 0.307)	− 0.013 (− 0.168, 0.168)	− 0.365 (− 0.578, − 0.128)***
Health event (2-month lag)	0.061 (− 0.099, 0.247)	− 0.009 (− 0.174, 0.174)	− 0.028 (− 0.263, 0.199)
Health event (3-month lag)	0.078 (− 0.114, 0.269)	0.008 (− 0.196, 0.194)	0.053 (− 0.206, 0.303)
Health event (4-month lag)	− 0.127 (− 0.330, 0.080)	− 0.017 (− 0.228, 0.176)	0.105 (− 0.149, 0.387)

***p < 0.001

**Fig. 3:**
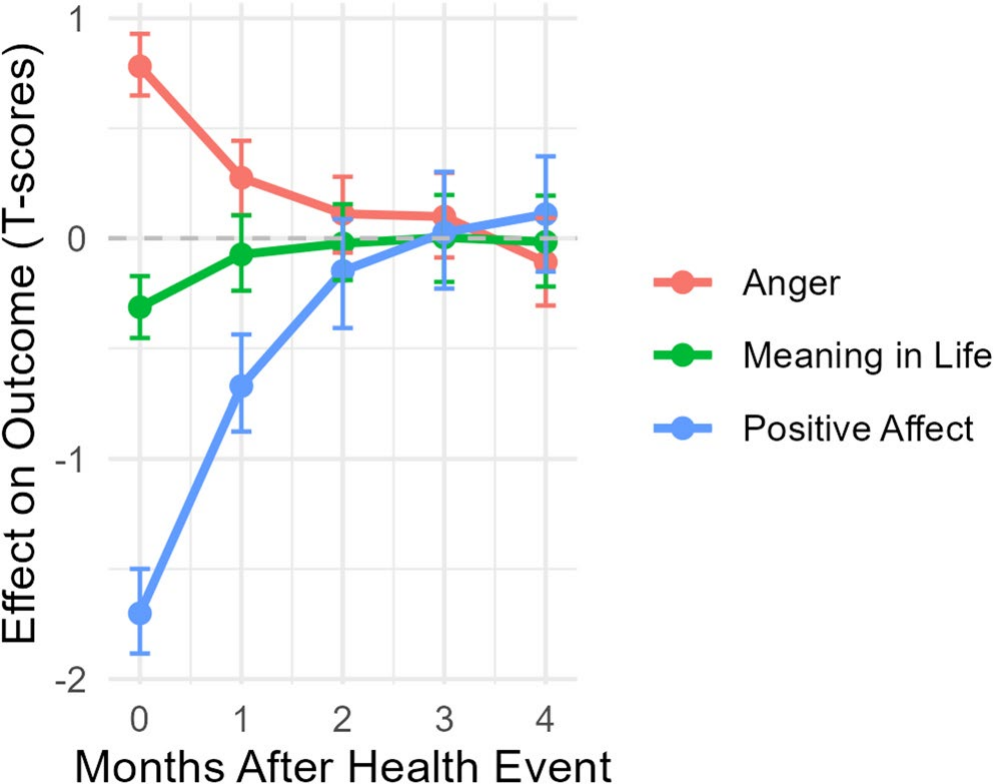
Impulse response functions of monthly PROMIS scores based on an acute health event. Error bars represent 95% credible intervals

To illustrate the magnitude and duration of PROMIS responses to a health event, we computed impulse response functions (IRFs) based on posterior estimates from the time-series model (Fig. 3). IRFs estimate the expected change in outcomes following the event, taking into account the immediate and lagged effects of all variables in the model. Anger showed a moderate immediate increase of nearly 1 T-score point, with effects persisting up to 2 months after the event due to lagged effects and autoregressive carryover. Positive affect exhibited the most pronounced immediate decline—over 1.5 T-score points—with IRFs returning to near zero by month 3. Meaning in life showed a more modest immediate decrease of approximately one-third of a T-score, with no evidence of lagged effects into subsequent months.

We also examined whether responses to health events varied across demographic groups by estimating a model that included interactions between the event indicator and all between-person covariates (age, gender, race, education, and number of chronic illnesses). As shown in Supplementary Table S7, most interaction effects were small and nonsignificant, indicating broadly similar event-related changes across groups. The one exception was that higher education significantly attenuated the increase in anger following a health event.

**Fig. 4:**
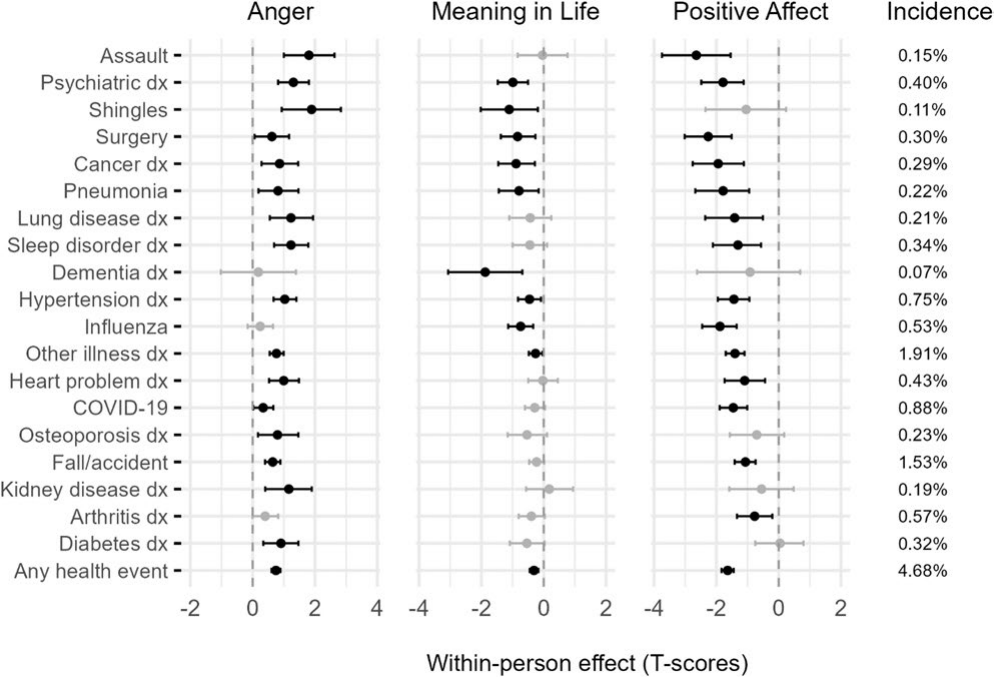
Concurrent within-person effects of specific health events on monthly PROMIS scores. Error bars represent 95% credible intervals. Effects shown in black are statistically significant (*p* < 0.05); effects shown in grey are not. Incidence values reflect the percentage of person-months during which each event was reported. *dx* = diagnosis

Supplemental analyses examined immediate (same-month) responses of PROMIS scores to each individual health event (Fig. 4). Anger increased significantly in response to 16 of the 19 events, with the strongest effects observed for assault (b = 1.81, 95% CI [1.00, 2.63]), being diagnosed with a psychiatric or emotional disorder (b = 1.30, 95% CI [0.82, 1.81]), and contracting shingles (b = 1.89, 95% CI [0.93, 2.83]). Meaning in life decreased significantly following 9 events, most notably after a diagnosis of dementia, senility, or other serious memory impairment (b = − 1.88, 95% CI [− 3.07, − 0.69]). Positive affect was significantly reduced by 14 events, with the largest declines associated with assault (b = − 2.64, 95% CI [− 3.75, − 1.54]), surgery or joint replacement (b = − 2.26, 95% CI [− 3.02, − 1.51]), and a cancer diagnosis (b = − 1.94, 95% CI [− 2.76, − 1.12]).

## Discussion

This study demonstrates that PROMIS CATs can be administered monthly in a large, population-based panel while maintaining strong psychometric properties. Three-item CATs achieved high measurement precision across most of the score range, and intraclass correlations indicated moderate rank-order stability in individual differences over time—supporting the feasibility of repeated PROMIS monitoring in general population research. Although reliabilities of 0.90 or higher have been documented for longer PROMIS CATs and are especially valuable for individual-level clinical monitoring [2], shorter CATs like those used here are well-suited for population-based research. Importantly, we show that these brief measures are sensitive to stable individual characteristics and to acute health disruptions and underlying chronic disease burden.

Consistent with earlier findings, we observed that greater disease burden with more chronic conditions was associated with lower average QoL levels [6]. A novel contribution of the present study is the demonstration that chronic conditions are also linked to greater fluctuation and instability in QoL over time. Monthly assessments allowed us to quantify meaningful individual differences in within-person variability—an aspect of QoL rarely examined in health research. Although increased within-person variability has been recognized as a signal of diminished psychological functioning in emotion research [11] and symptom tracking (e.g., pain) [32], its absence from most QoL studies may obscure important indicators of health vulnerability and disease impact. Our findings underscore the importance of capturing both average levels and intraindividual variability in self-reported PROMIS scores—an approach uniquely enabled by densely repeated assessments in monthly population surveys. These findings raise concerns about relying on single assessments in populations with worse health, where emotional wellbeing may fluctuate more. While our primary aim was to model variability as a meaningful psychological feature, elevated variability among those with multiple conditions suggests that one-time measurements may miss important aspects of their experience.

PROMIS scores were also responsive to acute health events, with most events showing immediate effects, particularly on anger and positive affect. In contrast, meaning in life appeared less reactive to acute disruptions, suggesting it may function as a more stable, trait-like aspect of psychological (or eudaimonic) well-being—potentially buffered by enduring values or broader beliefs that are less susceptible to transient events. Prior studies have demonstrated the sensitivity of PROMIS measures to acute health events and medical procedures, including increases in pain interference and behavior following hernia surgery, and heightened fatigue during chemotherapy [13]. However, such evidence has typically come from small or highly selected patient samples. In contrast, our findings demonstrate that PROMIS CATs detect meaningful real-world disruptions in QoL even in a large, population-based panel.

Effects of acute health events were generally short-lived, dissipating over the course of one to two months. The rapid attenuation may reflect natural recovery or psychological resilience among individuals, consistent with research showing that emotional reactions to stressors often subside quickly unless compounded by chronic strain or inadequate coping resources [33]. The magnitude of these effects—typically 1 to 3 T-score points—corresponds to small effect sizes by conventional benchmarks (e.g., Cohen’s *d* ≈ 0.10–0.30). While modest, these changes are broadly consistent with thresholds for minimally important change in PROMIS measures, which are often estimated in the range of 2 to 6 points depending on domain and context [34]. Future work should examine which individuals return to baseline quickly versus those who exhibit longer-term declines. Our moderation analyses offer preliminary evidence that educational attainment may modestly buffer anger responses to acute health events.

Notably, different PROMIS domains were differentially affected by specific health events, highlighting the domain-specific consequences of various life disruptions. Anger increased most strongly following assault, the diagnosis of a psychological disorder, and contracting shingles. These findings align with prior research showing that anger is a frequent response to interpersonal victimization and trauma, including assault, due to violations of personal safety and autonomy [35]. Similarly, diagnoses of psychiatric conditions have been associated with elevated anger, potentially reflecting reactions to stigma and emotional turmoil [36]. The pronounced increase in anger following shingles—a condition triggered by reactivation of the varicella-zoster virus and often linked to stress-related immune suppression—may reflect the emotional toll of physical pain and uncertainty, which can provoke frustration and irritability [37].

Positive affect was most adversely impacted by assault, surgery, and cancer diagnosis. Reductions in positive affect following assault have been well documented, with emotional trauma contributing to immediate downturns in mood [35, 38]. Similarly, major biomedical events can sharply reduce positive affect, as individuals grapple with fear, uncertainty, and the anticipated burden of treatment and recovery [39]. Finally, meaning in life showed the strongest decline following diagnoses related to cognitive impairment such as dementia. Such conditions often erode individuals’ sense of autonomy, social connectedness, and long-term purpose, contributing to existential distress [40].

Several limitations should be considered. First, the observational design of this study precludes causal inference. While our models accounted for temporal dependencies and included relevant covariates, unmeasured confounding cannot be ruled out. Second, information on chronic conditions and monthly health events was based on self-report, which may be subject to reporting inaccuracies and recall bias. Third, we focused on only three PROMIS domains—each assessed using a 3-item CAT per wave—to minimize participant burden across 13 monthly assessments. Although these short CATs achieved strong reliability across much of the score range, the use of longer CATs (e.g., 4–7 items) may have yielded greater measurement precision and enhanced sensitivity to smaller or subtler changes. Expanding both the number of domains and the number of items per domain could improve coverage and sensitivity to change, but must be weighed against the practical constraints of administering repeated assessments in large-scale panel studies [41].

## Conclusions

This study shows that brief PROMIS CATs can be administered monthly while maintaining strong psychometric properties. This high-frequency approach enables the simultaneous assessment of both average levels and temporal dynamics of emotional wellbeing. By capturing fluctuations linked to chronic health conditions and acute health events, this method offers a richer picture of how health shapes everyday experience. These findings support the integration of PROMIS measures into longitudinal population health research.

## Supplementary Information

Below is the link to the electronic supplementary material.

Supplementary Material 1

## Data Availability

The de-identified data are available for registered users on the Understanding America Study website (https://uasdata.usc.edu/index.php).
