# Supplementary material for: Tracking quality of life-related outcomes in the U.S. population with monthly PROMIS computerized adaptive testing
Source: Qual Life Res. 2026 Jun 6;35(7):176. doi: 10.1007/s11136-026-04279-9 (PMC13242437; doi:10.1007/s11136-026-04279-9)
Supplement: Supplementary file 1 — Supplementary Material 1 [file 11136_2026_4279_MOESM1_ESM.docx]

**Supplementary online appendix**

**Tracking Quality of Life in the U.S. Population
with Monthly PROMIS Computerized Adaptive Testing**

Quality of Life Research

Junghaenel, D.U.^1,2^, Schneider, S.^1,2^, Stone, A.A.^1,2^, Orriens, B.^2^, Gutsche, T.^2^, Darling, J.^2^,
Perez-Arce, F.^2^, Hayden, O.^2^, Kapteyn, A.^2^

^1^Center for Economic & Social Research, University of Southern California, Los Angeles, CA USA

^2^Department of Psychology, University of Southern California, Los Angeles, CA, USA

Corresponding author email: [junghaen@usc.edu](mailto:junghaen@usc.edu)

**Table 1.** Sample characteristics of respondents and nonrespondents

|  | Analyzed respondents ^a^ (N = 12,231) | Nonrespondents ^b^ (N = 3,898) | Test for difference |
| --- | --- | --- | --- |
| Age |  |  |  |
| Mean (*SD*) years | 50.94 (*SD* = 16.31) | 46.63 (*SD* = 16.76) | *t* = 14.26, *p* <.001 |
| 18-34 years | 2287 (18.74%) | 1118 (28.78%) | χ^2^(3) = 213.06, *p* < .001 |
| 35-49 years | 3579 (29.32%) | 1153 (29.68%) |  |
| 50-64 years | 3389 (27.76%) | 930 (23.94%) |  |
| 65+ years | 2952 (24.18%) | 684 (17.61%) |  |
| Female | 7482 (61.19%) | 2298 (58.95%) | χ^2^(1) = 6.18, *p* = .01 |
| Race/Ethnicity |  |  |  |
| Non-Hispanic White | 7296 (59.72%) | 2060 (52.89%) | χ^2^(2) = 56.86, *p* < .001 |
| Hispanic | 1933 (15.82%) | 707 (18.15%) |  |
| Other or mixed | 2989 (24.46%) | 1128 (28.96%) |  |
| Education |  |  |  |
| Mean (*SD*) years | 14.73 (*SD* = 2.61) | 14.67 (SD = 2.65) | *t* = 1.21, *p* = .23 |
| High school or less | 2536 (20.74%) | 837 (21.48%) | χ^2^(2) = 3.45, *p* = .17 |
| Some college | 4107 (33.58%) | 1346 (34.54%) |  |
| Bachelor’s degree or   higher | 5586 (45.68%) | 1714 (43.98%) |  |
| Annual Household Income |  |  |  |
| Less than $50,000 | 4613 (37.80%) | 1487 (38.25%) | χ^2^(2) = 0.48, *p* = .79 |
| $50,000-$99,999 | 3609 (29.57%) | 1128 (29.01%) |  |
| $100,000 or more | 3981 (32.62%) | 1273 (32.74%) |  |

*Note:* ^a^ Analyzed respondents are those completing more than 1 survey wave. ^b^ nonrespondents are those who were invited and completed 0 or 1 survey waves.

**Table S2. Comparison of standard errors (T-score metric) and reliability between the 3-item CAT and the shortest available PROMIS fixed short form in each domain.**

| **PROMIS Domain** | **Method** | **Mean SE (T)** | **Median SE (T)** | **Mean Reliability** | **Median Reliability** |
| --- | --- | --- | --- | --- | --- |
| **Meaning in Life** |  |  |  |  |  |
|  | CAT (3 items) | 3.84 | 3.4 | 0.85 | 0.88 |
|  | Fixed short form  (4 items) | 4.59 | 4.08 | 0.79 | 0.83 |
| **Anger** |  |  |  |  |  |
|  | CAT (3 items) | 3.96 | 3.85 | 0.84 | 0.85 |
|  | Fixed short form  (5 items) | 3.66 | 3.47 | 0.87 | 0.88 |
| **Positive Affect** |  |  |  |  |  |
|  | CAT (3 items) | 3.62 | 3.28 | 0.87 | 0.89 |
|  | Fixed short form  (15 items) | 2.27 | 2.07 | 0.95 | 0.96 |

*Note.* CAT standard errors reflect empirical EAP-based estimates from the administered 3-item CATs. Fixed-form standard errors were computed by applying the calibrated PROMIS item parameters to each participant’s observed theta estimate, converting test information to the T-score metric, and computing reliability as $1-\text{SE}_{T}^{2}/100$. Fixed forms included 4 items (Meaning in Life), 5 items (Anger), and 15 items (Positive Affect). For Positive Affect, the 15-item SF is substantially longer than the 3-item CAT and, as expected, yielded higher precision. However, administration of a 15-item SF at monthly intervals is not feasible in the present study due to respondent burden. Although less precise, the 3-item CAT retained meaningful information across a wide score range, supporting its use as a pragmatic compromise between measurement precision and feasibility.

**Table S3. Item usage frequencies for each 3-item CAT, showing the proportion of administrations in which each item was selected, the mean (SD) final theta estimate of participants who received the item, and item parameter estimates from the calibrated PROMIS models.**

|  |  | **Person parameter** | | **Item parameters** | |
| --- | --- | --- | --- | --- | --- |
| **Item index** | **Frequency (%)** | **Mean theta** | ***SD* of thetas** | **Slope** | **Mean threshold** |
| *Anger* | | | | | |
| 2 | 14.86 | -0.61 | 0.48 | 2.35 | 0.66 |
| 17 | 22.10 | -0.35 | 0.56 | 2.50 | 0.76 |
| 15 | 33.33 | 0.12 | 0.86 | 2.99 | 0.96 |
| 8 | 18.49 | 0.71 | 0.62 | 2.82 | 1.59 |
| 20 | 11.22 | 1.05 | 0.53 | 2.79 | 1.93 |
| *Meaning in life* | | | | | |
| 24 | 9.04 | -1.30 | 0.60 | 3.00 | -1.09 |
| 28 | 23.56 | -0.75 | 0.77 | 3.02 | -1.08 |
| 17 | 17.52 | -0.26 | 0.69 | 2.97 | -0.88 |
| 37 | 33.33 | -0.22 | 1.08 | 3.14 | -1.13 |
| 2 | 9.77 | 1.06 | 0.50 | 2.97 | -0.93 |
| 4 | 6.77 | 1.33 | 0.27 | 2.67 | -0.95 |
| *Positive affect* | | | | | |
| 5 | 17.63 | -1.26 | 0.65 | 3.23 | -0.91 |
| 28 | 28.50 | -0.63 | 0.96 | 3.44 | -0.94 |
| 33 | 33.33 | -0.27 | 1.25 | 3.50 | -0.99 |
| 32 | 15.71 | 0.84 | 0.71 | 3.26 | -0.28 |
| 1 | 4.83 | 1.85 | 0.19 | 2.89 | -0.06 |

*Note.* Mean theta refers to the participant’s final theta estimate from the CAT administration. In each domain, one item appears with a frequency of ~33% because it was administered first at every measurement occasion, reflecting the fixed CAT initialization at theta = 0. The remaining items show clear adaptive behavior: lower-difficulty items were administered at lower theta levels, and higher-difficulty items at higher theta levels.

**Table S4. Unadjusted intraclass correlations (ICCs) for each PROMIS domain, computed separately for participants with and without chronic conditions.** ICCs reflect the proportion of total variance attributable to stable between-person differences. These descriptive estimates do not adjust for demographic differences between groups (e.g., age, education, sex, race), which are addressed in the covariate-adjusted location-scale model (see Table 2 in the main text).

| **PROMIS Domain** | **Group** | **Between-Person Variance** | **Within-Person Variance** | **ICC** |
| --- | --- | --- | --- | --- |
| **Anger** | No chronic condition | 50.09 | 26.33 | 0.66 |
|  | With chronic condition | 48.54 | 23.11 | 0.68 |
| **Meaning in Life** | No chronic condition | 90.46 | 25.14 | 0.78 |
|  | With chronic condition | 91.60 | 23.10 | 0.80 |
| **Positive Affect** | No chronic condition | 106.15 | 49.35 | 0.68 |
|  | With chronic condition | 109.65 | 43.24 | 0.72 |

**Note.** Because individuals with chronic conditions differ demographically from those without (e.g., older, lower education), raw ICCs partially reflect these compositional differences. In covariate-adjusted location-scale models, chronic conditions are consistently associated with *greater* within-person variability (see Table 2).

**Table S5:** Multilevel regressions (95% credible intervals) of average levels and within-person variability in monthly PROMIS scores on demographic characteristics and chronic illnesses.

| Between-person predictors | Anger | Meaning in life | Positive affect |
| --- | --- | --- | --- |
| Average PROMIS level |  |  |  |
| Intercept  (no chronic condition) | 50.76***  (50.61, 50.92) | 48.70***  (48.47, 48.93) | 48.51***  (48.325 48.76) |
| Age (years) | -.169***  (-.178, -.160) | .083***  (.071, .096) | .087***  (.073, .100) |
| Female | -.949***  (-1.200, -.692) | .930***  (.570, 1.301) | .482*  (.081, .913) |
| White race | .852***  (.540, 1.150) | -2.169***  (-2.551, -1.753) | -1.076***  (-1.541, -.624) |
| Education (years) | -.171***  (-.220, -.127) | .158***  (.099, .228) | .262***  (.186, .332) |
| Diabetes | 1.080*** (.758, 1.398) | -.998***  (-1.445, -1.533) | -1.817***  (-2.365, -1.309) |
| Cancer | .370  (-.072, .803) | -.632*  (-1.257, -.048) | -.932**  (-1.668, -.258) |
| Lung disease | 1.766***  (1.190, 2.349) | -2.510***  (-3.288, -1.719) | -3.088***  (-4.009, -2.232) |
| Heart disease | .445  (.028, .852) | -.982***  (-1.576, -.409) | -.901**  (-1.544, -.227) |
| Stroke | .632 (-.165, 1.416) | -1.082*  (-2.277, .004) | -1.006  (-2.207, .241) |
| Arthritis | 1.354*** (1.043, 1.673) | -1.539***  (-2.001, -1.074) | -2.520***  (-3.001, -2.017) |
| Within-person variability |  |  |  |
| Intercept  (no chronic condition) | 2.767*** (2.735, 2.796) | 2.188***  (2.129, 2.252) | 3.210***  (3.164, 3.260) |
| Age (years) | -.016***  (-.018, -.015) | -.015***  (-.018, -.011) | -.011***  (-.013, -.008) |
| Female | .127***  (.081, .174) | .166***  (.079, .255) | .295***  (.217, .362) |
| White | -.312***  (-.371, -.257) | -.226***  (-.345, -.125) | -.025  (-.113, .064) |
| Education (years) | -.038***  (-.047, -.028) | -.109***  (-.126, -.090) | -.016*  (-.030, -.001) |
| Diabetes | .122***  (.064, .179) | .195***  (.069, .316) | .052  (-.040, .147) |
| Cancer | .084*  (.007, .169) | .161  (-.004, .328) | .119  (-.012, .257) |
| Lung disease | .113*  (.007, .225) | .135  (-.080, .321) | -.018  (-.186, .139) |
| Heart disease | .104**  (.023, .179) | .132  (-.013, .294) | -.023  (-.143, .092) |
| Stroke | .079  (-.066, .232) | .161  (-.128, .438) | .172  (-.064, .411) |
| Arthritis | .061*  (.004, .115) | .117*  (.002, .230) | .082  (-.010, .180) |

*Note:* **p* <.05,***p* < .01, ****p* < .05

**Table S6:** Within-person effects (95% credible intervals) of health events on monthly PROMIS scores, restricted to the subgroup of respondents (N= 3,545) reporting at least one event over the 13 month period.

| Within-person predictors | Anger | Meaning in life | Positive affect |
| --- | --- | --- | --- |
| Autocorrelation AR(1) | .174 (.162, .186)*** | .199 (.187, .212)*** | .192 (.179, .204)*** |
| Health event (same month) | .788 (.636, .929)*** | -.309 (-.449, -.164)*** | -1.703 (-1.908, -1.489)*** |
| Health event (1-month lag) | .115 (-.043, .282) | .000 (-.169, .162) | -.345 (-.584, -.118)*** |
| Health event (2-month lag) | .042 (-.104, .244) | .017 (-.144, .192) | .013 (-.222, .266) |
| Health event (3-month lag) | .065 (-.104, .244) | .022 (-.168, .204) | .090 (-.170, .339) |
| Health event (4-month lag) | -.141 (-.343, .056) | -.016 (-.198, .215) | .137 (-.128, .422) |

*Note*: ****p*<.001.

**Table S7.** Moderation of within-person effects of health-events by demographic characteristics and chronic conditions. Results from multilevel time-series models including interaction terms between the within-person event indicator and between-person moderators.

|  | Event x moderator estimate (95% credible interval) | | |
| --- | --- | --- | --- |
| Between-person moderator | Anger | Meaning in life | Positive affect |
| Age (years) | -.002 (-.012, .007) | -.005 (-.160, .006) | -.015 (-.030, .002) |
| Female | -.078 (-.381, .150) | .076 (-.295, .408) | .440 (-.017, .930) |
| White race | -.090 (-.451, .330) | -.172 (-.612, .329) | .064 (-.525, .701) |
| Education (years) | -.096 (-.156, -.037)*** | .046 (-.015, .098) | .045 (-.032, .118) |
| Number of chronic conditions | -.004 (-.162, .140) | .072 (-.099, .213) | .169 (-.069, .367) |

*Note*: ****p*<.001.
